# Double zero tillage and foliar phosphorus fertilization coupled with microbial inoculants enhance maize productivity and quality in a maize–wheat rotation

**DOI:** 10.1038/s41598-022-07148-w

**Published:** 2022-02-24

**Authors:** M. N. Harish, Anil K. Choudhary, Sandeep Kumar, Anchal Dass, V. K. Singh, V. K. Sharma, T. Varatharajan, M. K. Dhillon, Seema Sangwan, V. K. Dua, S. D. Nitesh, M. Bhavya, S. Sangwan, Shiv Prasad, Adarsh Kumar, S. K. Rajpoot, Gaurendra Gupta, Prakash Verma, Anil Kumar, S. George

**Affiliations:** 1 ICAR–Indian Agricultural Research Institute, New Delhi, 110 012 India; 2 ICAR–Central Potato Research Institute, Shimla, Himachal Pradesh 171 001 India; 3 ICAR–National Bureau of Plant Genetic Resources, New Delhi, 110012 India; 4 ICAR–Central Research Institute for Dryland Agriculture, Hyderabad, 500 059 India; 5 CCS Haryana Agricultural University, Hisar, Haryana 125 004 India; 6 CSA University of Agriculture & Technology, Kanpur, Uttar Pradesh 208 002 India; 7 University of Agricultural & Horticultural Sciences, Shivamogga, Karnataka 577 204 India; 8 ICAR-National Bureau of Agriculturally Important Microorganisms, Kushmaur, Mau, Uttar Pradesh 275 103 India; 9 Institute of Agricultural Sciences, Banaras Hindu University, Varanasi, Uttar Pradesh 221 005 India; 10 ICAR–Indian Grassland and Fodder Research Institute, Jhansi, Uttar Pradesh 284 003 India; 11 ICAR-National Dairy Research Institute, Karnal, Haryana 132 001 India; 12 Farm Science Centre, GAD Veterinary and Animal Sciences University, Tarn Taran, Punjab 143 412 India; 13 Farm Science Centre, ICAR-Indian Institute of Horticultural Research, Gonikoppal, Karnataka 571213 India

**Keywords:** Biochemistry, Plant sciences, Environmental sciences

## Abstract

Maize is an important industrial crop where yield and quality enhancement both assume greater importance. Clean production technologies like conservation agriculture and integrated nutrient management hold the key to enhance productivity and quality besides improving soil health and environment. Hence, maize productivity and quality were assessed under a maize–wheat cropping system (MWCS) using four crop-establishment and tillage management practices [FBCT–FBCT (Flat bed–conventional tillage both in maize and wheat); RBCT–RBZT (Raised bed–CT in maize and raised bed–zero tillage in wheat); FBZT–FBZT (FBZT both in maize and wheat); PRBZT–PRBZT (Permanent raised bed–ZT both in maize and wheat], and five P-fertilization practices [P_100_ (100% soil applied-P); P_50_ + 2FSP (50% soil applied-P + 2 foliar-sprays of P through 2% DAP both in maize and wheat); P_50_ + PSB + AM-fungi; P_50_ + PSB + AMF + 2FSP; and P_0 _(100% NK with no-P)] in split-plot design replicated-thrice. Double zero-tilled PRBZT–PRBZT system significantly enhanced the maize grain, starch, protein and oil yield by 13.1–19% over conventional FBCT–FBCT. P_50_ + PSB + AMF + 2FSP, integrating soil applied-P, microbial-inoculants and foliar-P, had significantly higher grain, starch, protein and oil yield by 12.5–17.2% over P_100_ besides saving 34.7% fertilizer-P both in maize and on cropping-system basis. P_50_ + PSB + AMF + 2FSP again had significantly higher starch, lysine and tryptophan content by 4.6–10.4% over P_100_ due to sustained and synchronized P-bioavailability. Higher amylose content (24.1%) was observed in grains under P_50_ + PSB + AMF + 2FSP, a beneficial trait due to its lower glycemic-index highly required for diabetic patients, where current COVID-19 pandemic further necessitated the use of such dietary ingredients. Double zero-tilled PRBZT–PRBZT reported greater MUFA (oleic acid, 37.1%), MUFA: PUFA ratio and P/S index with 6.9% higher P/S index in corn-oil (an oil quality parameter highly required for heart-health) over RBCT-RBCT. MUFA, MUFA: PUFA ratio and P/S index were also higher under P_50_ + PSB + AMF + 2FSP; avowing the obvious role of foliar-P and microbial-inoculants in influencing maize fatty acid composition. Overall, double zero-tilled PRBZT–PRBZT with crop residue retention at 6 t/ha per year along with P_50_ + PSB + AMF + 2FSP while saving 34.7% fertilizer-P in MWCS, may prove beneficial in enhancing maize productivity and quality so as to reinforce the food and nutritional security besides boosting food, corn-oil and starch industry in south-Asia and collateral arid agro-ecologies across the globe.

## Introduction

Under the aegis of United Nations Sustainable Development Goals (SDGs), there is an urgent need to focus both on food and nutritional quality enhancement for eradication of all types of hunger and malnutrition by 2030 especially in under-developed countries^1^. We already know that rice–wheat cropping system (RWCS), a major system in south-Asia in general and India in particular, is a major contributor to the food and nutritional security of the region^2,3^. However, intensive agriculture practices under RWCS especially in the Indo-Gangetic Plains Region (IGPR) coupled with intensive conventional tillage^4,5^, sole use of chemical fertilizers^6–8^, over-exploitation of groundwater^9^, and in situ crop residue burning^10,11^; has led to stagnation in productivity with impaired quality, sub-soil compaction, soil health deterioration, groundwater depletion and gradual degradation of natural resource-base^11,12^. The escalating labour, capital, and energy requirements^4^ coupled with receding groundwater table (~ 0.30–0.40 m year^−1^)^13^, erratic rainfall pattern and intermittent droughts^5^, has further triggered the chronic fatigue in RWCS in south-Asian IGPR for over last three decades^5,11^. Rice and wheat crops’ residue burning has also long been a major cause of air pollution releasing huge gaseous emission in northern India^10,14^, impairing soil and human health and environment^15^. To deter these ill-effects, crop diversification and conservation agriculture (CA) are two viable options^4,16^, over the policy backed conventional RWCS^17^. Bringing *National Policy for Management of Crop Residues*^18^ in India is again a timely effort which stresses upon *in-situ* residue management through CA and other sustainable residue management methods^19^. Hence, research priorities integrating clean production technologies (CPTs) viz. CA, best nutrient management practices and crop diversification should be set-up to avert these production- and resource vulnerabilities in IGPR^17,20,21^. Overall, this study heeds to the SDGs of the United Nations (with respect to land degradation neutrality and land restoration) from exploitation to the sustainable use of resources^22,23^, and soil health management^24,25^; so as to end hunger**, **achieve food security and improved nutrition, and promote sustainable agriculture as per SDG2^26^. The climate-resilient conservation agriculture which follows minimal soil disturbance, crop residue retention and crop rotations^6,27^, have been advocated to significantly improve the soil organic matter and soil health in holistic manner besides enhanced resource-use efficiency and crop yields in the vulnerable agro-ecologies across the globe^8,21,28–35^. Since, maize is one of the important cereal crops in south-Asia after rice and wheat, with the consumption of ~ 39.4 Mt maize grains in the region where India alone consumes ~ 24 Mt maize grains^36^. Hence, in order to safeguard the food security of millions of south-Asian families, maize farming tailored with CA practices followed in maize–wheat cropping system (MWCS) may prove as viable alternative to diversify the RWCS and boosting the productivity while concurrently conserving the soil, environment and natural resources^21,27,37^.

Globally, maize (*Zea mays* L.) is grown in ~ 193.7 m ha area producing ~ 1147.6 Mt grains with an average yield of 5.92 t ha^−1^ ^36^. Alone in India, maize is grown on ~ 9.2 m ha area producing 27.8 Mt grains but with poor productivity ~ 3.05 t ha^−1^ ^36^, and quality^38^. Maize is popularly known as queen of the cereals because of its high yield potential and wider adaptability to diverse agro-ecologies^39^. Maize is a vital crop for food and nutritional security in world’s poorest regions in Asia, Africa and Latin America^1,40^. Worldwide, maize is consumed in ~ 94 developing countries comprising > 4.5 billion people where it supplies ~ 30% of total calorie needs. Maize grain is a good source of high quality starch^41^; while its oil contains essential polyunsaturated fatty acids (PUFAs) that are highly beneficial in the management of cardiovascular diseases due to their vital role in blood cholesterol regulation and lowering of elevated blood pressure^38,42^. Maize is a versatile industrial crop processed into various food and non-food products viz. starch, vegetable oil, sweeteners, beverages, glue, alcohol and bioethanol, etc. Alone in India, ~ 20% of total maize consumption is utilized for non-food industrial product development, ~ 14% of which is solely utilized in starch manufacturing for pharmaceutical, textile, paper and food industry uses^43^. Likewise, higher amylose content in maize grains is a beneficial trait as it contains resistant starch (RS) type-2 with low glycemic index^44^, which is high demand in food industry for diabetic patients^45^. Current COVID-19 pandemic has further necessitated the management of this major comorbidity factor (diabetes) using such dietary ingredients^46^. For meeting global edible oil demands, maize is again a vital alternative containing ~ 3–4% oils in maize germ, an oil-rich part of maize kernel^47,48^. Corn oil is a rich source of linoleic acid (essential fatty acid), which is one of the two essential acids necessary for the integrity of the skin, cell membranes and immune system and for synthesis of eicosanoids necessary for reproductive, cardiovascular, renal, gastrointestinal functions and resistance to the diseases besides being highly effective in lowering the serum cholesterol primarily low-density-lipoprotein cholesterol^49^. Overall, maize grains with higher starch, oil and protein content are in high demand in food and non-food industry^43,50^. However, lysine and tryptophan are the deficient amino acids in cereals like maize which are essential for making the building blocks of human body. The crop productivity and quality can be increased through appropriate best plant nutrition^17^, bio-fortification approaches^51^, and agronomic practices like CA^33,52,53^ besides using breeding tools^54^. Hence, maize productivity and quality enhancement through agronomic approaches assumes utmost importance in curtailing the hunger and malnutrition besides promoting its industrial usages^55^. As, ~ 73% of total global maize area is located in developing world, hence, improved productivity and quality traits through low-cost CPTs may open new vistas for maize growers and agri-entrepreneurs to fetch higher prices for quality maize produce in food and industrial sectors.

Phosphorus (P) is one of the most important nutrient elements which plays an important role in enhancing the productivity and quality while influencing various plant processes like energy storage and transfer, photosynthesis, root growth, flowering, seed setting and seed yield, etc.^56–58^. Due to poor native-P status, low solubility and low efficiency ~ 10–20% across the majority of global arable soils, the P is a critical nutrient that greatly limits plant growth, yield and quality^57,59^. In order to improve the productivity, quality and P-use efficiency (PUE) in this high nutrient requiring crop due to its high yield potential, devising efficient P management strategies with integration of soil applied P-fertilizers, biofertilizers and innovative approaches like foliar-P fertilization that too under CA based systems, may assume utmost importance. Alluvial soils in Indian IGPR are characterized as most fertile soils but now majority of them are diagnosed with low soil-P status^60^. The P-fertilizers are already very costly and the most of the soil applied-P gives low PUE with fate of native and applied-P being fixed as Ca and Mg phosphate in alkaline soils of IGPR^60^. Phosphorus-solubilizing bacteria (PSB) and AM-fungi also hold great potential in solubilization and mobilization of native and applied-P^51,61^. Foliar P-fertilization has also shown positive influence on crop productivity and quality in many crops^58,62^. Thus, foliar-P fertilization along with microbial inoculants may prove as a low-cost CPT in nutrient exhaustive crops like maize to harness higher yield with better quality and PUE besides saving soil applied-P. However, the impacts of conservation agriculture coupled with this innovative P-management strategy integrating soil applied P, foliar-P and microbial inoculants in maize–wheat cropping system are yet to be evaluated with respect to maize productivity and quality parameters (starch, protein, amino acid and fatty acid composition) that too under South-Asian semi-arid climate. Overall, the CPTs like conservation agriculture along with foliar-P fertilization may enhance both productivity and quality to augment its safe food and industrial uses besides improving soil health and environment. However, no systematic research work has been carried-out till date to assess the impact of such climate-resilient CPTs especially CA and the innovative foliar-P fertilization on quality parameters of maize in a semi-arid agro-ecology. Therefore, this study assessed the impacts of the CA based crop establishment and tillage management (CETM) and microbial inoculants’ imbedded P-fertilization practices on maize yield and grain quality under MWCS so as to scale-up the food and nutrition security under the precept of United Nations SDGs, besides augmenting its safe food and industrial uses in blooming food, starch and corn-oil industry in south-Asia.

## Results

### Maize grain yield

**Figure 1:**
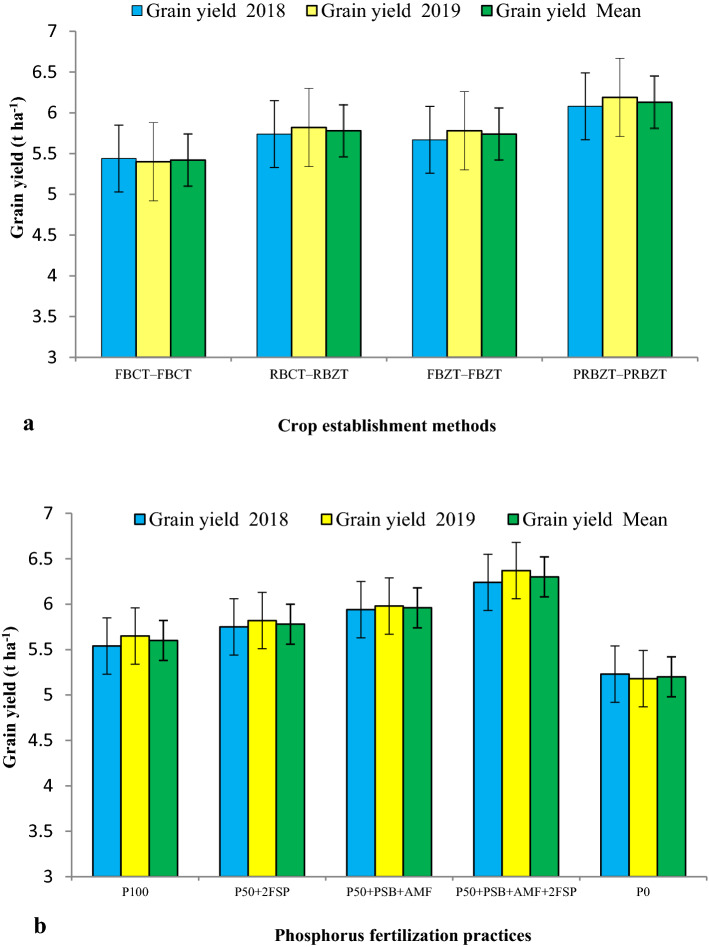
Influence of (**a**) CETM and (**b**) P-fertilization practices on grain yield (t ha^−1^) of maize under MWCS. The vertical bars represent LSD_0.05_ values.

Maize grain yield was significantly (*p* < 0.05) influenced by the crop establishment and tillage management practices (CETMs) as well as P-fertilization practices (PFPs) during both years (Fig. 1). The double zero-tilled permanent raised-beds with crop residue retention of 6 t ha^−1^ per year under treatment PRBZT–PRBZT in MWCS resulted in significantly higher mean maize grain yield (6.13 t ha^−1^) by 6.4, 5.7 and 13.1% over RBCT–RBZT, FBZT–FBZT and FBCT–FBCT. The integration of 50% P + PSB + AMF + 2FSP, a combination of soil applied-P, microbial inoculants and the two foliar-P sprays (2% DAP), observed significantly (*p* < 0.05) higher grain yield (6.3 t ha^−1^) by 5.4, 8.3, 11.3 and 17.5% over P_50_ + PSB + AMF, P_50_ + 2FSP, P_100_ and P_0_, respectively. However, P_50_ + 2FSP, P_50_ + PSB + AMF and P_100_ treatments were statistically at par with each other. The interaction effects between the CETMs and PFPs were found significant in the current study (Supplementary Table S1). On an average, PRBZT–PRBZT and P_50_ + PSB + AMF + 2FSP exhibited ~ 13.1 and 11.3% higher maize grain yield over their respective counterpart treatments FBCT–FBCT (conventional-tilled FB system both in maize and wheat) and the P_100_ (100% soil applied-P), respectively (Fig. 1).

### Starch content and starch yield

The CETMs exhibited higher magnitude of starch content (65.1–67.2%) in maize grains under CA plots (PRBZT–PRBZT, FBZT–FBZT, RBCT–RBZT) compared to 64.4% in the conventional-tilled FB system (FBCT–FBCT) (Table 1). Likewise, maize under raised-beds (PRBZT–PRBZT, RBCT–RBZT) exhibited higher starch content (~ 66.2%) compared to ~ 64.9% under flat-beds (FBZT–FBZT, FBCT–FBCT) irrespective of the tillage practices.

**Table 1:** Influence of CETM and P-fertilization practices on grain starch content (%), starch yield (kg ha^−1^), protein content (%) and protein yield (kg ha^−1^) in maize under MWCS.

Treatment	Starch (%)			Starch yield (kg ha^−1^)			Protein content (%)			Protein yield (kg ha^−1^)		
	2018	2019	Mean	2018	2019	Mean	2018	2019	Mean	2018	2019	Mean
**CETM practices**												
FBCT–FBCT	64.5	64.2	64.4	3511	3494	3503	8.85	8.91	8.88	481.4	483.0	482.2
RBCT–RBZT	65.5	64.6	65.1	3774	3836	3805	9.18	9.22	9.20	527.8	538.2	533.0
FBZT–FBZT	65.5	65.3	65.4	3752	3798	3775	9.12	9.38	9.25	520.6	543.3	531.9
PRBZT–PRBZT	67.4	67.0	67.2	4113	4202	4157	9.17	9.50	9.33	558.3	589.5	573.9
CD (*p* = 0.05)	NS	NS	NS	362	438	307	NS	0.30	0.3	34.0	49.4	29.1
**P–fertilization practices**												
P_100_	66.3	65.9	66.1	3681	3767	3724	9.10	9.35	9.23	505.2	528.0	516.6
P_50_ + 2FSP	67.0	67.1	67.0	3854	3914	3884	9.30	9.62	9.46	535.9	560.5	548.2
P_50_ + PSB + AMF	64.9	65.3	65.1	3855	3906	3881	9.23	9.43	9.33	548.2	563.6	555.9
P_50_ + PSB + AMF + 2FSP	68.6	69.2	68.9	4288	4413	4350	9.48	9.70	9.59	591.9	619.8	605.6
P_0_	62.1	61.0	61.5	3260	3163	3211	8.28	8.15	8.22	428.9	421.2	425.0
CD (*p* = 0.05)	4.3	4.2	4.1	354	358	321	0.49	0.43	0.45	30.9	32.8	24.4
**Interaction**	NS	NS	NS	NS	NS	NS	NS	NS	NS	NS	S	NS

CD values indicate the critical difference at *p* = 0.05.

Among PFPs, significantly (*p* < 0.05) higher starch content (68.9%) were obtained by applying P_50_ + PSB + AMF + 2FSP followed by P_50_ + 2FSP (67.1%) and least under P_0_ (61.6%). Despite of non-significant effect of CETMs on starch content, the starch yield was significantly (*p* < 0.05) influenced by both CETMs and PFPs with significantly (*p* < 0.05) higher values under PRBZT–PRBZT (4157 kg ha^−1^), a double zero-tilled PRB system, and the P_50_ + PSB + AMF + 2FSP (4350 kg ha^−1^), a combination of soil applied-P, microbial inoculants and the two foliar-P sprays (Table 1). On an average, PRBZT–PRBZT and P_50_ + PSB + AMF + 2FSP reported ~ 18.6 and 16.8% higher starch yield over their respective counterpart treatments FBCT–FBCT and P_100,_ respectively (Table 1).

### Amylose and amylopectin content

**Figure 2:**
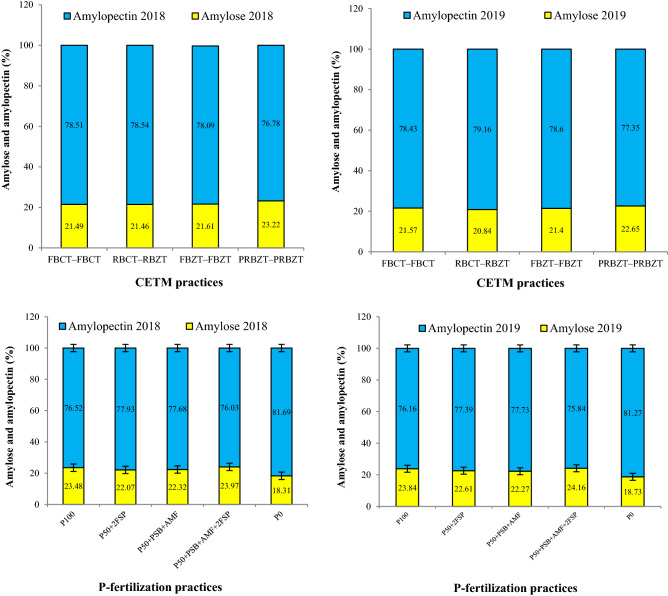
Influence of CETM and P-fertilization practices on grain amylose and amylopectin content of maize under MWCS. The vertical bars represent LSD_0.05_ values.

The CETMs did not show any significant influence on the amylose content while PFPs exhibited significant effect (*p* < 0.05) on the amylose content in maize grains during both years (Fig. 2). Under CETMs, highest amylose content (23%) were reported under PRBZT–PRBZT while other treatments exhibited ~ 21.2–21.5% amylose content. Integration of P_50_ + PSB + AMF + 2FSP resulted in significantly (*p* < 0.05) higher amylose content (24.1%) which was followed by P_100_, P_50_ + 2FSP, P_50_ + PSB + AMF and P_0_, respectively (Fig. 2). On an average, PRBZT–PRBZT and P_50_ + PSB + AMF + 2FSP exhibited ~ 6.5 and 1.7% higher amylose content over the FBCT–FBCT and P_100_, respectively. The PFPs again exhibited significant effect (*p* < 0.05) on amylopectin content while CETMs did not show any significant influence on amylopectin content during both years (Fig. 2). The amylopectin followed the reverse trend as that of amylose content both for CETMs and PFPs. Highest amylopectin content was achieved under RBCT–RBZT (78.9%) and least under PRBZT–PRBZT (77.1%). The P_0_ exhibited significantly (*p* < 0.05) higher amylopectin content (81.5%) while P_50_ + PSB + AMF + 2FSP exhibited least values (75.9%).

### Protein content and protein yield

The CETMs had non-significant influence on protein content in maize grains during first year; however the effects were significant (*p* < 0.05) during second year (Table 1), where CA-based CETMs (PRBZT–PRBZT, FBZT–FBZT, RBCT–RBZT) had higher protein content (9.2–9.33%) compared to FBCT–FBCT (8.88%). Maize grown on raised-beds (PRBZT–PRBZT, RBCT–RBZT) exhibited higher protein content compared to flat-beds (FBZT–FBZT, FBCT–FBCT) during both years. Double zero-tilled PRBZT–PRBZT system produced ~ 5.1% higher protein content compared to conventional-tilled FBCT–FBCT system. Among PFPs, P_50_ + PSB + AMF + 2FSP exhibited significantly (*p* < 0.05) higher protein content (9.59%) which was followed by P_50_ + 2FSP with lowest magnitude under P_0_ (9.46%) during the study. The integration of P_50_ + PSB + AMF + 2FSP enhanced the protein content by ~ 3.9% over the recommended PFP (P_100_). The CETMs and PFPs both had significant (*p* < 0.05) influence on protein yield following the similar trend as that of grain yield where PRBZT–PRBZT and P_50_ + PSB + AMF + 2FSP both produced significantly superior protein yield during both years (Table 1). However, the interaction effects between the CETMs and PFPs for the protein yield were found to be significant only during second year (Supplementary Table S2). On an average, PRBZT–PRBZT and P_50_ + PSB + AMF + 2FSP exhibited ~ 19 and 17.2% higher protein yield over their respective counterpart treatments viz*.* FBCT–FBCT and P_100_, respectively (Table 1).

### Lysine and tryptophan content

The CETMs did not have any significant effect on the lysine content in maize grains, however, the raised-bed CETMs viz. PRBZT–PRBZT and RBCT-RBZT produced higher lysine content over their flat-bed counterpart CETMs viz. FBZT–FBZT and FBCT–FBCT, respectively (Table 2). The PRBZT–PRBZT observed ~ 1.2% higher lysine content over the FBCT–FBCT. The P_50_ + PSB + AMF + 2FSP reported significantly higher lysine content (2.74 g kg^−1^ dry matter) which was followed by P_100_, P_50_ + 2FSP, P_50_ + PSB + AMF and P_0_, respectively (Table 2). Integration of P_50_ + PSB + AMF + 2FSP exhibited ~ 6.6% higher lysine content over 100% soil applied-P. The CETMs again didn’t show any significant influence on tryptophan content, although, double zero-tilled PRBZT–PRBZT and FBZT–FBZT treatments reported comparatively higher tryptophan content over the conventional-tilled FBCT–FBCT system. Among PFPs, P_50_ + PSB + AMF + 2FSP had significantly higher tryptophan content by ~ 10.4% over P_100_. In general, tryptophan followed similar trend as that of protein content both for CETMs and PFPs in current study (Table 2).

**Table 2:** Influence of CETM and P-fertilization practices on lysine (g per kg dry matter), tryptophan content (µg g^−1^) and grain-P uptake (kg ha^−1^) of maize under MWCS.

Treatment	Lysine content (g per kg dry matter)			Tryptophan (µg g^−1^)			P uptake by grains (kg ha^−1^)		
	2018	2019	Mean	2018	2019	Mean	2018	2019	Mean
**CETM practices**									
FBCT–FBCT	2.45	2.44	2.44	0.64	0.65	0.64	14.92	15.45	15.18
RBCT–RBZT	2.48	2.46	2.47	0.64	0.66	0.65	16.30	16.77	16.53
FBZT–FBZT	2.44	2.48	2.46	0.66	0.66	0.66	14.54	17.35	15.94
PRBZT–PRBZT	2.48	2.47	2.47	0.65	0.67	0.66	18.28	19.03	18.65
SEm ±	0.02	0.03	0.03	0.02	0.02	0.02	0.60	0.38	0.41
CD (*p* = 0.05)	NS	NS	NS	NS	NS	NS	2.01	1.31	1.41
**P–fertilization practices**									
P_100_	2.55	2.59	2.57	0.65	0.67	0.67	14.78	16.52	15.65
P_50_ + 2FSP	2.47	2.50	2.48	0.72	0.73	0.72	16.46	17.81	17.13
P_50_ + PSB + AMF	2.45	2.42	2.44	0.66	0.69	0.68	17.18	17.29	17.24
P_50_ + PSB + AMF + 2FSP	2.72	2.75	2.74	0.74	0.75	0.74	18.73	20.98	19.86
P_0_	2.11	2.06	2.09	0.45	0.43	0.44	12.88	13.14	13.01
SEm ±	0.06	0.06	0.06	0.02	0.02	0.02	0.72	0.57	0.51
CD (*p* = 0.05)	0.18	0.17	0.18	0.04	0.05	0.04	2.06	1.64	1.47
**Interaction**	NS	NS	NS	NS	NS	NS	S	S	S

CD values indicate the critical difference at *p* = 0.05.

### Oil content and oil yield

Effect of CETMs on corn-oil content was found non-significant (Table 3). However, double zero-tilled PRBZT–PRBZT (4.83%) and FBZT–FBZT (4.74%) reported higher corn-oil content over the single-crop based zero-tilled RBCT–RBZT (4.65%) and no-tilled FBCT–FBCT (4.63%). Likewise, maize under raised-beds (PRBZT–PRBZT, RBCT–RBZT) exhibited higher oil content over their counterpart flat-bed CETMs (FBZT–FBZT, FBCT–FBCT) irrespective of the tillage followed. On an average, double zero-tilled PRBZT–PRBZT system realized ~ 4.3% higher oil content over the CT based FBCT–FBCT. Among PFPs, significantly (*p* < 0.05) higher oil content was obtained by applying P_50_ + PSB + AMF + 2FSP (4.9%) followed by P_100_ (4.84%) and least under P_0_ (4.41%). The P_50_ + PSB + AMF + 2FSP enhanced the oil content by ~ 1.2 and 11.1% over P_100_ and P_0_, respectively. Oil yield was significantly influenced by both CETM and PFPs with significantly (*p* < 0.05) higher magnitude under PRBZT–PRBZT (297.2 kg ha^−1^) and P_50_ + PSB + AMF + 2FSP (309.4 kg ha^−1^) to the tune of ~ 18.1 and 14.3% over their respective counterpart treatments viz. FBCT–FBCT and P_100_ (Table 3).

**Table 3:** Influence of CETM and P-fertilization practices on oil content (%) and oil yield (kg ha^−1^) of maize under MWCS.

Treatment	Oil content (%)			Oil yield (kg ha^−1^)		
	2018–19	2019–20	Mean	2018–19	2019–20	Mean
**CETM practices**						
FBCT–FBCT	4.62	4.64	4.63	251.3	252.0	251.7
RBCT–RBZT	4.63	4.66	4.65	266.6	272.3	269.4
FBZT–FBZT	4.73	4.75	4.74	269.3	274.2	271.8
PRBZT–PRBZT	4.81	4.86	4.83	292.7	301.7	297.2
CD (*p* = 0.05)	NS	NS	NS	25.09	18.56	15.00
**P–fertilization practices**						
P_100_	4.81	4.86	4.84	266.8	274.6	270.7
P_50_ + 2FSP	4.79	4.82	4.81	276.2	281.3	278.7
P_50_ + PSB + AMF	4.59	4.61	4.60	272.9	275.6	274.2
P_50_ + PSB + AMF + 2FSP	4.86	4.95	4.90	303.0	315.7	309.3
P_0_	4.43	4.40	4.41	231.2	228.0	229.6
CD (*p* = 0.05)	0.25	0.24	0.25	21.55	22.56	19.7
**Interaction**	NS	NS	NS	NS	NS	NS

CD values indicate the critical difference at *p* = 0.05.

### Fatty acid profiling

**Figure 3:**
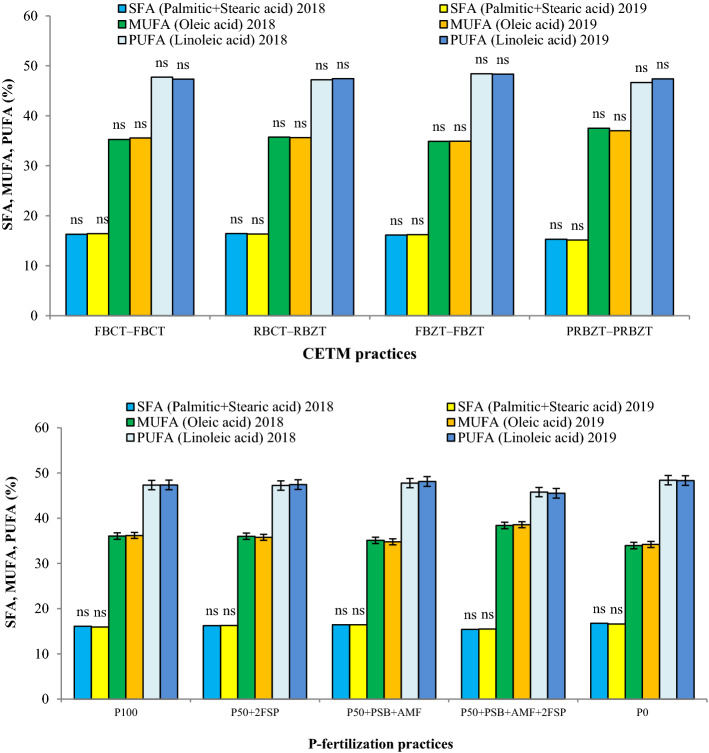
Influence of CETM and P-fertilization practices on fatty acid composition viz. SFA [Saturated fatty acid (Palmitic acid + Stearic acid)], MUFA [Mono unsaturated fatty acid (Oleic acid)] and PUFA [Poly unsaturated fatty acid (Linoleic acid)] in maize under MWCS. The vertical bars represent LSD_0.05_ values.

Effect of CETMs on fatty acid content in maize grain oil was found non-significant (Fig. 3). However, the saturated fatty acid (SFA) (Palmitic acid + Stearic acid) and poly unsaturated fatty acid (PUFA) (Linoleic acid) content, were higher under conventionally-tilled plots (RBCT–RBZT, FBCT–FBCT) compared to double zero-tilled plots (PRBZT–PRBZT, FBZT–FBZT). A reverse trend was observed for mono unsaturated fatty acid (MUFA) content (Oleic acid) where double zero-tilled CETMs (PRBZT–PRBZT, FBZT–FBZT) exhibited higher MUFA (Oleic acid) content over the conventionally-tilled RBCT–RBZT and FBCT–FBCT CETMs. The PRBZT–PRBZT exhibited highest MUFA (oleic acid) content (37.3%) while FBZT–FBZT had highest PUFA (Linoleic acid) content (48.4%). The PFPs showed significant effect on PUFA and MUFA composition except SFA (Palmitic acid + Stearic acid). The SFA and PUFA content were higher under P_0_ (16.7; 48.4%) and lowest under P_50_ + PSB + AMF + 2FSP (15.5; 45.7%), respectively; whereas MUFA content were higher under P_50_ + PSB + AMF + 2FSP (38.5%) and least under P_0_ (34.1%); exhibiting the obvious role of PFPs in influencing fatty acid concentration in corn-oil. No-P supply (P_0_) resulted in inhibitory effect on MUFA (Oleic acid) content and resulted in higher SFA (Palmitic and Stearic acid) and PUFA (Linoleic acid) content. Thus, MUFA and PUFA content differed significantly due to PFPs. The SFA and PUFA followed the trend of P_0_ > P_50_ + PSB + AMF > P_50_ + 2FSP > P_100_ with respective higher values (16.7; 48.4%) under P_0_ and lowest values (15.5; 45.7%) under P_50_ + PSB + AMF + 2FSP, respectively. The MUFA content showed reverse trend with higher values under P_50_ + PSB + AMF + 2FSP (38.5%) and least under P_0_ (34.1%). On an average, PRBZT–PRBZT and P_50_ + PSB + AMF + 2FSP exhibited ~ 5.2 and 6.5% higher MUFA (Oleic acid) content over their respective counterpart treatments viz. FBCT–FBCT and P_100_ (Fig. 3).

### Fatty acid ratios

**Figure 4:**
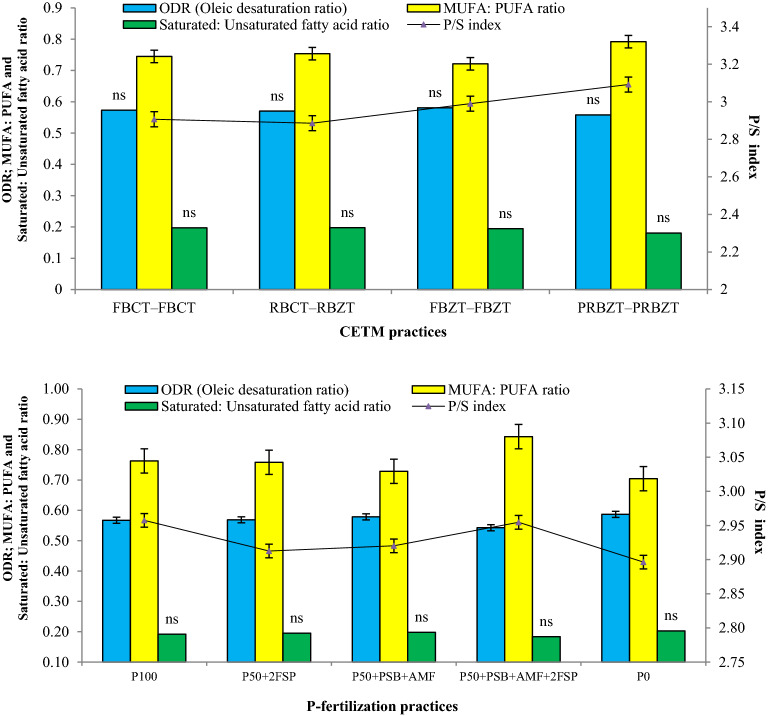
Influence of CETM and P-fertilization practices on various fatty acid ratios of maize under MWCS. The vertical bars represent LSD_0.05_ values.

Fatty acid ratios in corn-oil viz. oleic desaturation ratio (ODR), MUFA: PUFA ratio, SFA: unsaturated fatty acid ratio (SFA: UFA ratio) and P/S index showed considerable variations for CETMs and PFPs (Fig. 4). The ODR and SFA: UFA ratio didn’t show any significant differences while MUFA: PUFA ratio and P/S index exhibited significant differences under CETMs. Double zero-tilled PRBZT–PRBZT had highest MUFA: PUFA ratio (0.79) and P/S index (3.09) while other CETMs were statistically similar amongst them. The ODR, MUFA: PUFA ratio and P/S index responded positively and significantly (*p* < 0.05) to PFPs (Fig. 4). The P_0_ had highest ODR (0.59) but with least MUFA: PUFA ratio (0.70) and P/S index (2.9). The P_50_ + PSB + AMF + 2FSP had least ODR (0.54) but with highest MUFA: PUFA ratio while remaining PFPs were statistically similar amongst them for ODR and MUFA: PUFA ratio. Highest P/S index was found under P_100_ (2.96) followed by P_50_ + PSB + AMF + 2FSP, P_50_ + PSB + AMF and P_50_ + 2FSP, respectively. On an average, double zero-tilled PRBZT–PRBZT and soil-applied P_100_ exhibited ~ 6.9 and 2.1% higher P/S index in corn-oil over their respective counterpart treatments viz. RBCT–RBCT and P_0_ (Fig. 4).

### Maize grain P-uptake

The maize grain P-uptake followed the trend of PRBZT–PRBZT > RBCT–RBZT > FBZT–FBZT > FBCT–FBCT with significantly higher values under double zero-tilled PRBZT–PRBZT system (18.7 kg ha^−1^) (Table 2). Among PFPs, integrated use of P_50_ + PSB + AMF + 2FSP led to significantly (*p* < 0.05) higher grain P-uptake, though it was statistically similar to P_100_ following the trend P_50_ + PSB + AMF + 2FSP > P_50_ + PSB + AMF > P_50_ + 2FSP > P_100_ > P_0_ (Table 2). On an average, double zero-tilled PRBZT–PRBZT and P_50_ + PSB + AMF + 2FSP exhibited ~ 22.9 and 26.9% higher grain P-uptake over their respective counterpart treatments viz. FBCT–FBCT and P_100_.

### Correlation studies

**Figure 5:**
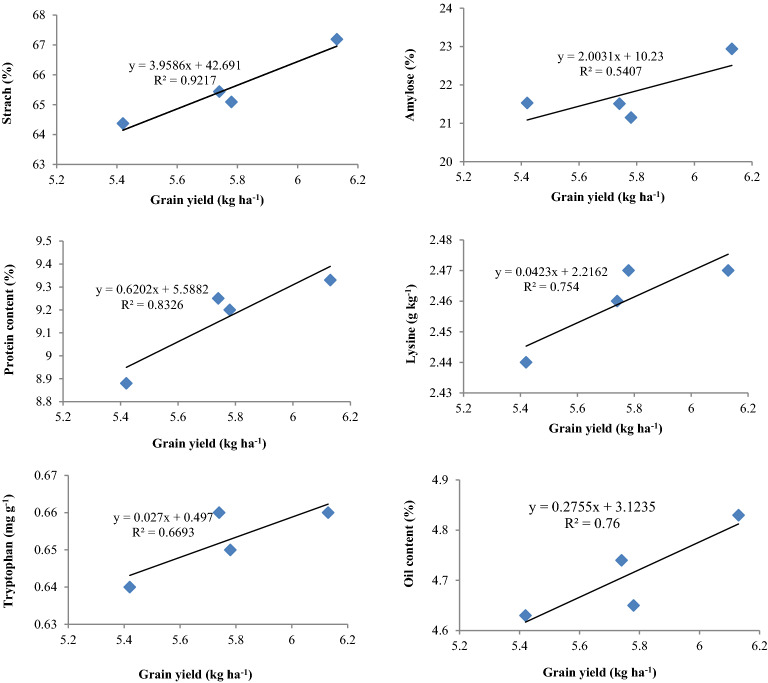
Correlation between grain yield and quality parameters of maize under CETM practices in maize under MWCS.

**Figure 6:**
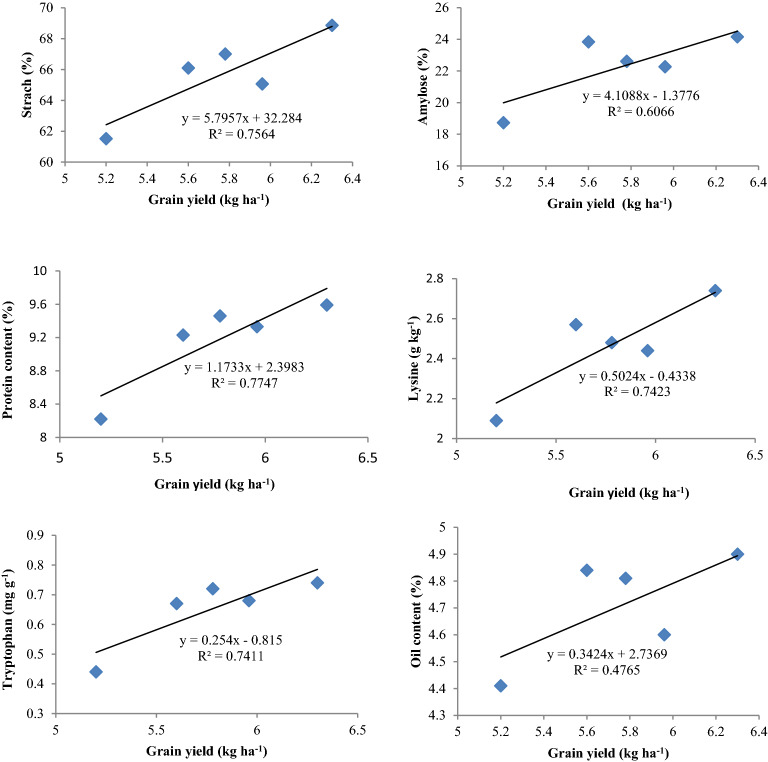
Correlation between grain yield and quality parameters of maize under P-fertilization practices in maize under MWCS.

**Figure 7:**
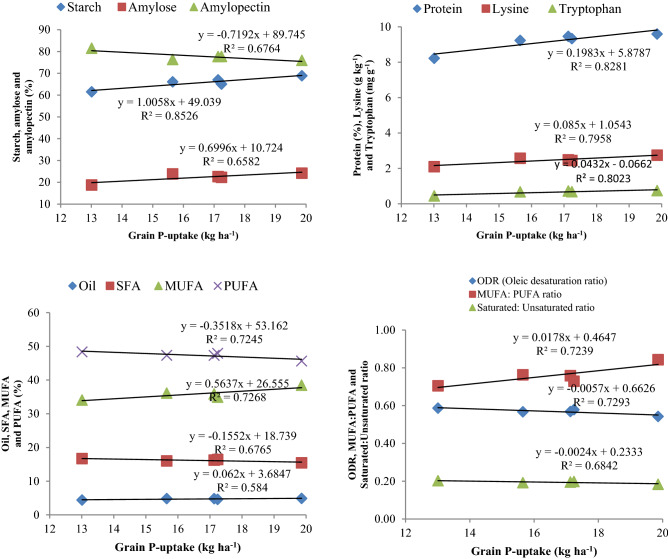
Correlation between grain P-uptake and quality parameters of maize under P-fertilization practices in maize under MWCS.

Starch content in maize grains showed positive correlation (*p* < 0.05) with grain yield both for CETMs (R^2^ = 0.921) and PFPs (R^2^ = 0.756) (Figs. 5, 6). Starch content again showed positive correlation (*p* < 0.05) with grain P-uptake (R^2^ = 0.852) under PFPs (Fig. 7). Amylose content had positive correlation (R^2^ = 0.54; 0.606), while amylopectin had negative association (R^2^ = − 0.54; − 0.635) with grain yield both for CETMs and PFPs, respectively (Figs. 5, 6). Amylose content again had positive association (R^2^ = 0.658) while amylopectin had negative correlation (R^2^ = − 0.676) with grain P-uptake under PFPs (Fig. 7). More to the point, protein content showed positive correlation with grain yield both under CETMs (R^2^ = 0.832) and PFPs (R^2^ = 0.774) as well as with grain P-uptake (R^2^ = 0.828) under PFPs (Figs. 5, 6, 7). Lysine (R^2^ = 0.754; 0.742) and tryptophan content (R^2^ = 0.669; 0741) had positive correlation with grain yield both for CETMs and PFPs, respectively (Figs. 5, 6).

Likewise, lysine (R^2^ = 0.795) and tryptophan content (R^2^ = 0.802) showed positive correlation with grain P-uptake under PFPs (Fig. 7). Oil content had strong positive association (R^2^ = 0.76) with grain yield under CETMs (Fig. 5), but a moderate positive correlation (R^2^ = 0.476) under PFPs (Fig. 6). Oil content showed a moderate positive correlation with grain P-uptake (R^2^ = 0.584) under PFPs (Fig. 7). Among various fatty acids, only MUFA had positive correlation (*p* < 0.05) with grain P-uptake (R^2^ = 0.726) while SFA (R^2^ = − 0.676) and PUFA (R^2^ = − 0.724) had negative relationship with grain P-uptake under PFPs (Fig. 7). Grain P-uptake and MUFA: PUFA ratio had a positive correlation (R^2^ = 0.723), whereas ODR (R^2^ = − 0.729) and SFA: UFA ratio (R^2^ = − 0.684) had negative correlation with grain P-uptake under PFPs (Fig. 7).

### Principal component analysis and clustered heatmap

**Figure 8:**
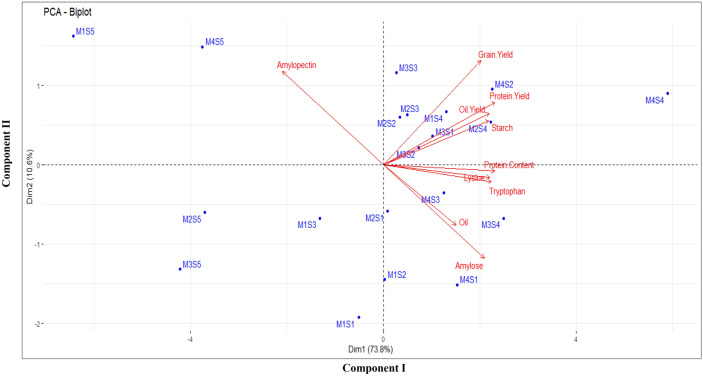
PCA biplots showing the effect of different treatment combinations of CETMs and PFPs on productivity and quality parameters of maize (pooled data).

**Figure 9:**
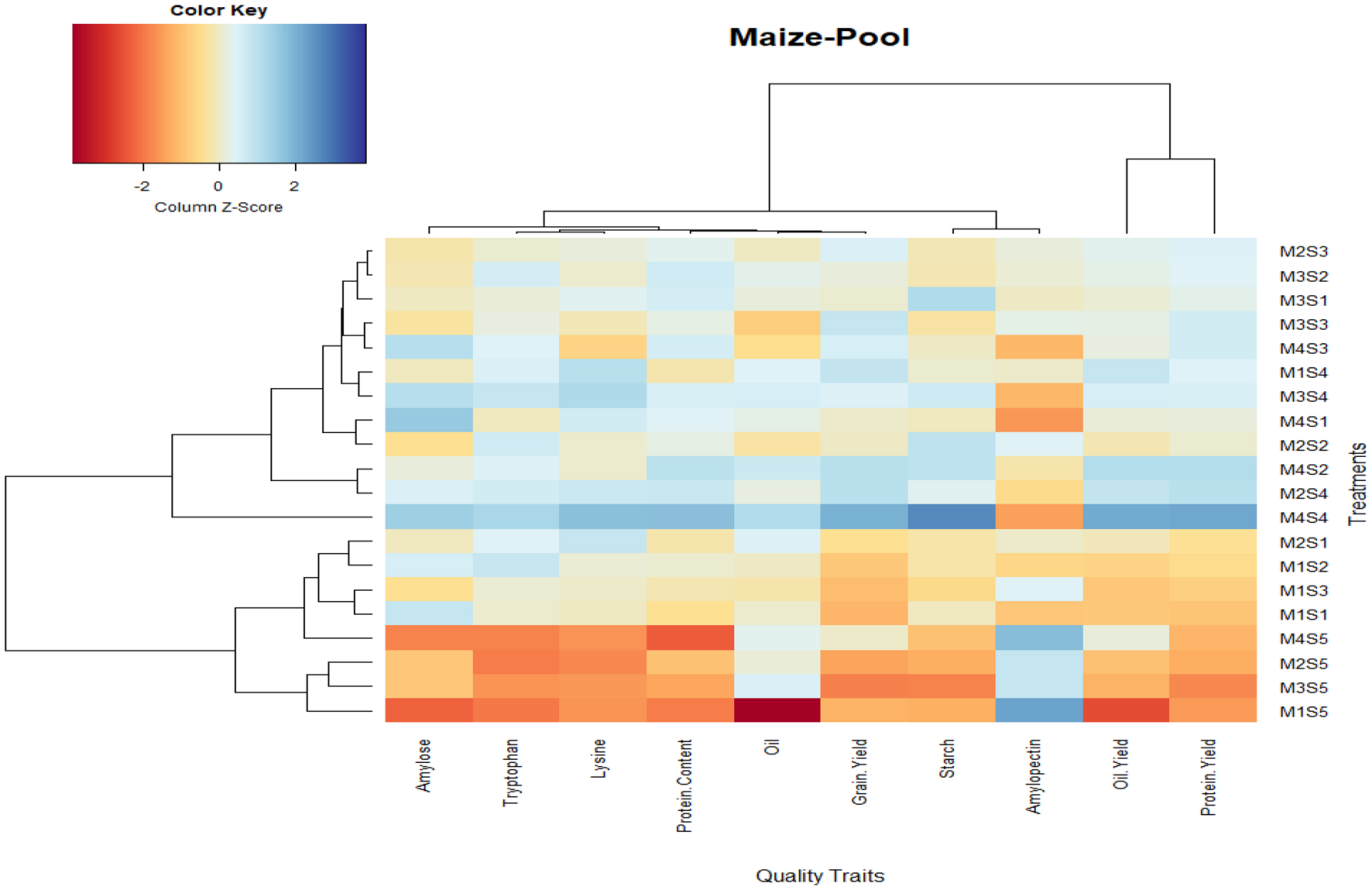
Biclustering heatmap analysis of maize productivity and the quality parameters at different treatment combinations of CETMs and PFPs in maize (pooled data), using R-software package ‘gplots’, Software version number ‘R package version 3.1.1’, Software URL https://CRAN.R-project.org/package=gplots.

Principal component analysis (PCA) revealed the differences in the composition of maize quality parameters under different combinations of CETMs and PFPs (*p* < 0.05). As shown in Fig. 8, all the treatment combinations clustered distinctly. Treatment combinations M_4_S_3_ (PRBZT–PRBZT coupled with P_50_ + PSB + AMF) and M_3_S_4_ (FBZT–FBZT coupled with P_50_ + PSB + AMF + 2FSP) exhibited higher amylose, protein, lysine, tryptophan and oil content. Likewise, M_4_ (PRBZT–PRBZT), M_3_ (FBZT–FBZT) and M_2_ (RBCT–RBZT) in combination with S_4_ (P_50_ + PSB + AMF + 2FSP), S_3_ (P_50_ + PSB + AMF) and S_2_ (P_50_ + 2FSP) had a positive correlation with both component one and component two, exhibiting increased grain yield, protein yield, oil yield and starch content. The conventionally-tilled M_1_ (FBCT–FBCT) along with S_5_ (P_0_) exhibited higher amylopectin content than other treatment combinations (Fig. 8). For a better understanding of the clustering pattern of grain yield and quality parameters across the treatment combinations; a biclustering heatmap was generated (Fig. 9). This heatmap showed that maize yield and quality parameters (except amylopectin) clustered closely and displayed an increase under CA based CETMs (M_4_, M_3_ and M_2_) in combination with the PFPs viz. S_4_ (P_50_ + PSB + AMF + 2FSP), S_3_ (P_50_ + PSB + AMF) and S_2_ (P_50_ + 2FSP). Most significant and remarkable shifts were found for amylopectin content where conventionally-tilled M_1_ clustered closer to the CA based CETMs (M_4_, M_3_ and M_2_) all supplied with no-P. On an average, CA based CETMs (M_4_ and M_3_) in combination with S_4_ (P_50_ + PSB + AMF + 2FSP) and S_2_ (P_50_ + 2FSP) showed higher similarity to each other and formed a cluster for maize yield and the majority of the quality parameters. The M_4_S_4_ (CA based PRBZT–PRBZT supplied with P_50_ + PSB + AMF + 2FSP) was proved as best treatment combination for realizing higher maize yield and quality parameters as tangibly evident from the heatmap (Fig. 9).

## Discussion

Diversifying the existing dominant RWCS towards viable alternative maize-based systems particularly the MWCS^16,21,35^, the conservation agriculture based CETMs (PRBZT–PRBZT; FBZT–FBZT) along with the appropriate P-fertilization practices (PFPs), could enhance and stabilize the yields besides improving soil health in long-run^17,63^, and more importantly the quality parameters of this potential food and industrial crop of south-Asia. The tillage and input-intensive RWCS in the IGPR of south-Asia is facing multiple production- and resource-vulnerabilities viz. exaggerating decline in crop productivity, groundwater table, input-use efficiencies and soil-health^5,11,17,21^. Henceforth, CA based MWCS has ample potential to combat these assailabilities besides resolving twin challenges of maize productivity and quality enhancement for ushering in food and nutritional security vis-à-vis augmenting industrial applications of this crop in south-Asia.

In this study, the CA-based double zero-tilled permanent raised-bed system (PRBZT–PRBZT) with crop residue retention at 6 t ha^−1^ per year in MWCS had significantly (*p* < 0.05) higher maize grain yield by 13.1% over the CT-based FBZT–FBZT, and by 5.7–6.4% over the double zero-tilled flat-bed system (FBZT–FBZT) and the single crop basis zero-tilled system in preceding wheat (RBCT–RBZT) across the years (Fig. 1). It could be associated with the positive impact of crop residue retention and zero-tillage on modulation of soil temperature^64,65^, improved water retention, infiltration and moisture conservation^29,66^, soil surface characteristics^5^, reduced crop-weed completion^67,68^, improved soil physico-chemical and biological properties^20,34,69^ and better water and nutrient usage^17,27,30^, resulting in better plant growth and yield^11^. Double ZT system provides better soil physical conditions due to less machine trafficking^65^, better seed germination and optimal seedling establishment due to avoidance of hard crust formation on soil surface, a characteristic feature of alluvial soils of IGPR^70^. Higher maize yield under PRB/RB plots (PRBZT–PRBZT, RBCT–RBZT) over the flat-bed CT and ZT plots may also be attributed to better root aeration and root anchorage in raised-beds^7,71^, least water stagnation during rains^27^, and better moisture conservation in rainless spans^72^. Crop residue retention and its slow decomposition enhance the SOC^6^ and soil moisture content^27^, both of which are ideal for favorable soil biological activities in ZT^20,73,74^, which eventually augment the nutrient bio-availability^10,75^ favoring growth and productivity^67,70,76^.

Since, P-fertilization directly influences the root growth and development which in turn improved the vegetative and reproductive growth vis-à-vis maize yield^57,77^. Integration of P_50_ + PSB + AMF + 2FSP, a combination of soil applied-P, microbial inoculants (PSB, AMF) and the two foliar-P sprays (2% DAP), had significantly (*p* < 0.05) higher grain yield by 11.3–17.5% over the soil applied-P_100_ and P_0_ (Fig. 1). Furthermore, the integrated use of P_50_ + PSB + AMF + 2FSP saved ~ 34.7% fertilizer-P over the soil applied-P_100_ both in maize alone and on cropping system basis in MWCS. In alkaline soils of semi-arid IGPR, soil applied-P reacts with calcium (Ca) and magnesium (Mg) ions to form Ca and Mg phosphates making P unavailable to plants^57^. Hence, 2 foliar-P sprays at knee-high and pre-tasseling stage of maize proved beneficial for P-absorption through foliage which enhanced the plant growth and photosynthetic activity leading to improved maize yield^58^. Foliar-P skip the P-fixation and leads to higher PUE^62^, which otherwise is an unavoidable fate of soil applied-P in alkaline and acidic soils^61^. Inoculation of maize grains with PSB and AMF along with 50% soil applied-P proved effective even over 100% soil applied-P due to improved P-availability and uptake owing to their synergistic effect on P-solubilization and mobilization of fixed native- and applied-P^51,78^. Furthermore, the AMF mycelia growth greatly enhances the root exploratory area (10–1000 folds), thus, helping in better nutrient and water acquisition^61,79^. Exudation of organic acids/chelating agents by AMF mineralizes the organic residues and manures to release inorganic nutrients with better phyto-availability^65,80^ besides enriching soil microbial diversity^7,8^, thus adding to better yields.

Phosphorus and potassium nutrition is vital for starch biosynthesis^81,82^. The CA based CETMs (PRBZT–PRBZT, FBZT–FBZT, RBCT–RBZT) had higher starch content (65.1–67.2%) compared to 64.4% in the CT based FBCT–FBCT (Table 1). This may be attributed to enhanced macro- and micronutrient availability especially P and K^30^, owing to residue decomposition with better nutrient-recycling especially K^10,83^, and mineralization and solubilization of native-and applied-P by the organic acids released from decomposing residues under ZT^28,84^. Likewise, maize under raised-beds (PRBZT–PRBZT, RBCT–RBZT) exhibited higher starch content (~ 66.2%) compared to ~ 64.9% under flat-beds (FBZT–FBZT, FBCT–FBCT) irrespective of tillage practices owing to better root aeration and anchorage for nutrients^71^ especially limiting nutrients like P^61^. Thus, ZT based CETMs enhanced the starch content over the conventional-tillage. Among PFPs, significantly (*p* < 0.05) higher starch (68.9%) was obtained by integrated use of P_50_ + PSB + AMF + 2FSP followed by P_50_ + 2FSP and least under P_0_. Here, foliar-P fertilization proved beneficial in higher P absorption by maize foliage and its assimilation which enhanced the starch content. Under P-deficiency, starch content decreases because of reduced ATP production in chloroplast resulting in reduced activity of ADPG enzyme, a key enzyme in starch metabolism; so the starch produced in chloroplast was unable to diffuse to cytoplasm as tri-phosphate, thus, resulting in reduced translocation of carbohydrates to grains^82^. Starch content showed positive correlation with grain yield both for CETMs and PFPs, owing to greater role of starch in grain biomass accumulation being influenced by both CETMs^30^, and P-fertilization^38^. The CETM practices again did not show any significant effect on amylose and amylopectin content in maize grains like starch content. Starch biosynthesis is mainly dependant on proteins present in the starch granules^82^, particularly the granule-bound starch synthase I protein (GBSSI) which is involved in amylose synthesis^85^. Hence, enhanced N-availability and protein content in ZT based CETMs might have enhanced the amylose content to some extend over CT based CETMs. Henceforth, higher amylose content under PRBZT–PRBZT and P_50_ + PSB + AMF + 2FSP may be associated to improved nutrient availability and acquisition^86–88^. Higher amylose content under PRBZT–PRBZT and 50% P + PSB + AMF + 2FSP, considered as a beneficial trait due to its lower glycemic-index, required by diabetic patients^45^. As, amylose and amylopectin together constitute the starch, hence, the treatments having higher amylose had a lower amylopectin content and vice-versa as evident from correlation studies and the heatmap clustering. Furthermore, amylose content and grain yield had positive correlation while amylopectin had negative correlation with yield both under CETMs and PFPs. Starch yield was significantly influenced by CETMs and PFPs due to higher grain yield under PRBZT–PRBZT and P_50_ + PSB + AMF + 2FSP treatments. In nutshell, PRBZT–PRBZT along with P_50_ + PSB + AMF + 2FSP proved beneficial to harness higher starch content, starch yield and amylose content; which can amplify the maize based food, starch and pharmaceutical industry in the south-Asia.

The ZT based CETMs had higher protein content over the conventional-tillage due to residue retention (3–6 t ha^−1^ per year) which on decomposition and mineralization enhanced the N-availability and uptake to synthesize amino acids and proteins^20,89,90^. Higher protein content in maize may also be associated with preferential deposition of *zein* protein over other endosperm proteins^89,91^. Protein yield was higher under ZT based CETMs compared to CT plots due to improved nutrient availability and soil health^92,93^, optimal soil moisture status and better root activities^88,94^. The PFPs had significant influence on protein content and protein yield owing to the vital role of P in protein biosynthesis and energy relations^81,82^. Furthermore, the P and N are found to have synergistic effect, thus, integrated use of P_50_ + PSB + AMF + 2FSP might have significantly enhanced the N uptake and assimilation^95^, leading to greater protein content and protein yield^96^. Nutritionally essential amino acids viz. lysine and tryptophan are highly important to improve maize grain quality^97^. Here, different CETMs had non-significant effect on lysine and tryptophan content. It may be strengthened with the fact that an increase in grain-N content as a result of improved N-availability is accompanied by decrease in the relative lysine content of grain proteins^98^. On the other hand, P indirectly influences the lysine content because when P-supply is reduced, it results in reduced grain yield but with increased grain-N concentration; thus, leads to reduced lysine content in grains^98^. Although under optimal P-fertilization, here P_50_ + PSB + AMF + 2FSP, the grain yield increases which results in reduced grain-N content due to dilution effect, which in turn, increases the lysine content under optimal or excess P-supply^99^. Henceforth, a similar pattern was observed for lysine content under PFPs in current study. Since, P and Zn are found to have antagonistic effects, so the P plays a vital role in tryptophan production^100^. The Zn is involved in various oxidation–reduction reactions^101^; thereby, Zn-deficiency leads to oxidation of auxins and reduction of tryptophan^102^. As, tryptophan is the precursor of auxins^97^, hence, tryptophan was higher under P_50_ + PSB + AMF + 2FSP over soil applied-P_100_; because of reduced Zn-uptake under soil applied-P_100_ compared to P_50_ + PSB + AMF + 2FSP, a combination of soil, microbial and foliar-P application which had an advantage over soil applied-P owing to reduced competition for Zn-uptake^100,103^. Positive correlation of protein, lysine and tryptophan content with the grain yield both under CETMs and PFPs, further emphasize the importance of ZT based CETMs and integrated use of P_50_ + PSB + AMF + 2FSP in enhancement of protein, lysine and tryptophan content as well as protein yield. Thus, deployment of such CPTs may altogether boost farm productivity and quality for better profitability of resource-poor south-Asian farmers and the maize based food, feed, and pharmaceutical industry in the region.

Different CETM practices had non-significant effect on oil content in maize grains although ZT based CETMs proved superior over conventional-tillage; where PRBZT–PRBZT had higher oil content due to crop residues decomposition^104^, which slowly released the essential nutrients (soluble-P and S-compounds) into rhizosphere which later became available to plants specifically during reproductive phase^105^. Sulfur (S) is a key element in chlorophyll formation, yield enhancement and oil synthesis^57^. Furthermore, S-concentration and S-uptake has a strong synergistic relationship with P in plants^57,106^. Positive correlation and heatmap clustering between oil content and grain P uptake under PFPs has further strengthens this fact. As per an estimate, the wheat straw of 2700 kg ha^−1^, on average add ~ 28 kg N, 4.5 kg P, 52 kg K and 6 kg S ha^−1^ under ZT system; on the other hand, this advantage may lack in CT system^107^. Hence, crop residues decomposition released both P and S while additional application of foliar-P augmented S uptake from the soil, thereby, enhancing the oil content. The P_50_ + PSB + AMF + 2FSP exhibited significantly higher oil content and oil yield over other PFPs which may be accrued to the fact that P directly participates in synthesis of oils, fats and phospholipids^108^, besides its vital role in S acquisition^57^. Better plant nutrition under CA based CETMs and integrated P-fertilization practices though caused a slight improvement in grain oil content in current study^109^, but harnessed greater oil production per unit area because of enhanced grain yield^38^. Positive correlation between oil content and maize grain yield has strongly established this relationship in current study. Hence, maize cultivation under PRBZT–PRBZT system along with P_50_ + PSB + AMF + 2FSP may lead to higher corn-oil productivity which may cut down the oil imports by the developing nations like India.

The ZT and CT based CETMs exhibited non-significant effect on the composition of fatty acids viz. saturated fatty acids (SFA), mono unsaturated fatty acids (MUFA) and poly unsaturated fatty acid (PUFA) like oil content. However, these fatty acids were greatly influenced by PFPs both under CT and ZT systems with pattern of fatty acid composition as PUFA > MUFA > SFA both under CETMs and PFPs^110^. With increase in P-supply, SFA (Palmitic acid + Stearic acid) content decreased while MUFA (Oleic acid) content increased considerably, again as an indicator of better oil quality^42^. These observations are in agreement with the findings of Ray^38^, who observed similar findings with respect to SFA and MUFA content while using higher doses of plant nutrients. The PUFA (linoleic acid) content were higher under P_0_ in current study; which further corroborate with the findings of Krueger^56^, who observed an increase in linoleic acid with the P-omission. In current study, the influence of different CETMs and PFPs on the status of individual fatty acid may not lead to definite conclusion about the overall fatty acid composition in corn-oil. Thus, various fatty acid ratios were estimated to draw logical conclusions. Among CETMs, double zero-tilled PRBZT–PRBZT had significantly higher MUFA: PUFA ratio and P/S index over CT plots which show better oil quality under ZT system owing to better N-supply encouraging carbon chain elongation in linoleic acid (PUFA) and oleic acid (MUFA)^111–113^. The P/S index is a vital factor among all parameters as it represents the nutritional value of edible oils^38^. Here, P/S index was found to be > 1.0 irrespective of CETMs and PFPs, which sufficiently indicated the better nutritional value of corn-oil with reduced tendency of deposition of lipids in the human body^114^. It was found that ZT based CETMs and P_50_ + PSB + AMF + 2FSP exhibited higher P/S index over the CT system and P_0_. It is reported that with an increase in unsaturation content and a degree in fatty acid, the susceptibility to oil oxidation increases; thus, releasing free radicals causing off-flavor and reduced nutritional quality^38^. On average, oleic acid is 25-times less vulnerable to oxidation compared to linoleic acid, while linoleic acid is 2-times less susceptible compared to linolenic acid because of an increase in bond association energy as compared to linolenic acid^115^. As, ZT based CETMs and P_50_ + PSB + AMF + 2FSP had higher oleic acid and lowest linoleic acid content, a positive sign for producing good quality corn-oil having less susceptibility to oxidation which may help in flourishing the corn-oil industry. Linolenic acid is susceptible to oxidation and causes adverse effect on human health like cardiovascular diseases and improper brain development^116,117^. As corn-oil contained negligible amount (< 1%) of linolenic acid in current study, hence, it would not exert any adverse effect on human health^38^. Under P_0_, higher oleic desaturation ratio (ODR) and lesser MUFA: PUFA ratio compared to other PFPs again point out a better quality corn-oil^105^. Higher ODR indicates better and longer shelf-life of corn-oil; while lower ODR inhibits the subsequent desaturation steps which lead to reduced linolenic acid content^112^. The P-fertilization considerably increased the ODR and MUFA: PUFA ratio under P_50_ + 2FSP, P_50_ + PSB + AMF and sole P_100_; but P_50_ + PSB + AMF + 2FSP showed slight reduction in ODR and an increase in MUFA: PUFA ratio may be due to enhanced P-availability over P_0_. The MUFA: PUFA ratio is directly linked with the oxidative stability and nutritional properties of the oil^118^, thus, indicating that optimal P-nutrition and the ZT system both may improve the oil quality due to sustained and synchronized P bio-availability throughout crop season. Significantly higher P/S index under P_50_ + PSB + AMF + 2FSP is another indicator of better nutritional value of edible maize oil^38^. The heatmap also demonstrated that optimal P-nutrition and ZT system proved highly promising in producing good quality corn-oil, a good indication for corn-oil industry to target health conscious clientele^114^.

The CETMs and PFPs showed significant influence on P uptake in maize grains with greater magnitude under PRBZT–PRBZT and P_50_ + PSB + AMF + 2FSP, owing to higher grain-P concentrations and maize yield in these treatments. Higher grain-P uptake in PRBZT–PRBZT is attributed to affirmative effects of crop residue retention which added substantial amount of nutrients including P in soil while improving soil physico-chemical and microbiological properties compared to CT plots^27,72,119^. The P-fertilization along with PSB and AMF vis-à-vis foliar-P had a significant influence on grain-P uptake as a result of optimal P bio-availability^61^, better root and shoot system^11^, enhanced native and applied-P solubilization and mobilization^65,120^, and foliar-P supplementation^58^; which collectively led to higher P uptake^121^. Thus, better the P-fertilization better is the P uptake by the crop and its subsequent accumulation in grains^62^. The P-fertilization in adequate amounts is essential for root and shoot development, seed formation and biochemical reactions viz., synthesis of proteins, oils and fats, phospholipids and energy relations, thus, it played a vital role in enhancing the maize quality^108^. That’s why, the quality parameters of maize viz., starch, protein, lysine, tryptophan, MUFA, and MUFA: PUFA ratio had positive correlation with grain-P uptake. Contrary to that, the amylopectin, PUFA, SFA, ODR and SFA: UFA ratio showed an inverse relationship with the grain-P uptake owing to complex interrelationships with their counterpart constituents^82,85,108^, and with varying P supplies as reported by various researchers^86–88,99,100^. The heatmap biclustering validated the superiority of CA-based PRBZT–PRBZT and FBZT–FBZT systems in combination with two PFPs viz. P_50_ + PSB + AMF + 2FSP and P_50_ + PSB + AMF in enhancing the grain, protein and oil yield as well as starch, amylose, lysine and tryptophan content; which demonstrate the sustainability of CA based crop management over the conventional agriculture while integrating P_50_ + PSB + AMF + 2FSP in MWCS. It is tangibly evident from the PCA analysis and heatmap biclustering that the M_4_S_4_, a combination of double zero-tilled PRBZT–PRBZT system in combination with P_50_ + PSB + AMF + 2FSP may prove highly sustainable for realizing higher maize grain yield and quality under a maize–wheat cropping system in a semi-arid agro-ecology. Thus, clean production technologies like double zero-tilled PRBZT–PRBZT along with P_50_ + PSB + AMF + 2FSP not only enhanced the maize yield significantly while saving ~ 34.7% fertilizer-P both in maize and MWCS, but they also augmented the maize quality parameters to reinforce the food and nutritional security besides boosting food, corn-oil and starch industry in the south-Asia.

## Conclusions

In order to safeguard the food and nutritional security of millions of south-Asian families concurrently conserving the soil, environment and natural resources, the application of clean production technologies (CPTs) like CA-based CETMs (PRBZT–PRBZT/FBZT–FBZT) that allows rapidly increases of yield and food quality should be a norm, not the exception. In our study, the production technology of the PRBZT–PRBZT/FBZT–FBZT along with integrated use of P_50_ + PSB + AMF + 2FSP in MWCS proved to be excelled in the maize yield and quality parameters. On average, double zero-tilled PRBZT–PRBZT system and P_50_ + PSB + AMF + 2FSP both significantly enhanced the maize grain, starch, protein and oil yield by 13.1–19% and 12.5–17.2%, over their respective counterpart treatments i.e. FBCT–FBCT and 100% soil applied-P (P_100_); while concurrently saving ~ 34.7% fertilizer-P both in maize (20.8 kg P_2_O_5_/ha) and on cropping system basis (41.6 kg P_2_O_5_/ha). Integrated use of P_50_ + PSB + AMF + 2FSP had significantly higher starch, amylose, protein, lysine, tryptophan and oil content by 1.2–10.4% over the 100% soil applied-P due to sustained and synchronized P bio-availability to the crop. The PRBZT–PRBZT had greater MUFA (oleic acid, 37.1%), MUFA: PUFA ratio (0.79) and P/S index (3.09). The ODR, MUFA: PUFA ratio and P/S index responded positively and significantly to P-fertilization practices. Double zero-tilled PRBZT–PRBZT system concurred with residue retention at 6 t ha^−1^ per year along with P_50_ + PSB + AMF + 2FSP while saving ~ 34.7% fertilizer-P in MWCS, proved as a potential clean production technology for enhancing the maize productivity and quality. Accordingly, deserves strong recommendation to augment maize yield and quality besides augmenting safe industrial uses in maize based industries, climate-resilience and farmers’ well-being in semi-arid IGPR in south-Asia and similar agro-ecologies across the globe.

## Materials and methods

### Experimental details and crop management

**Figure 10:**
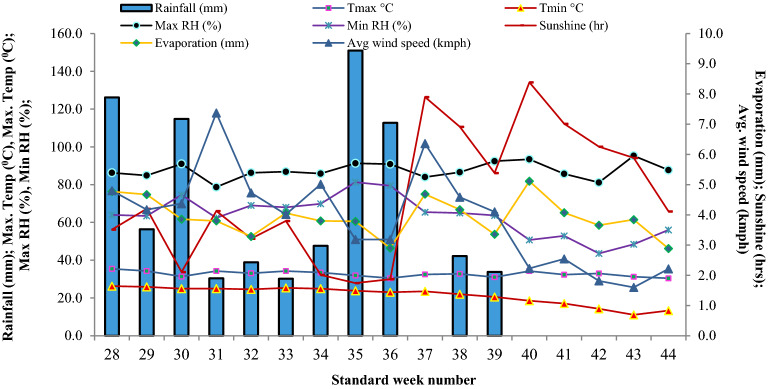
Weekly weather conditions during the cropping period of *Kharif* season maize in the Indo-Gangetic Plains, 2018.

**Figure 11:**
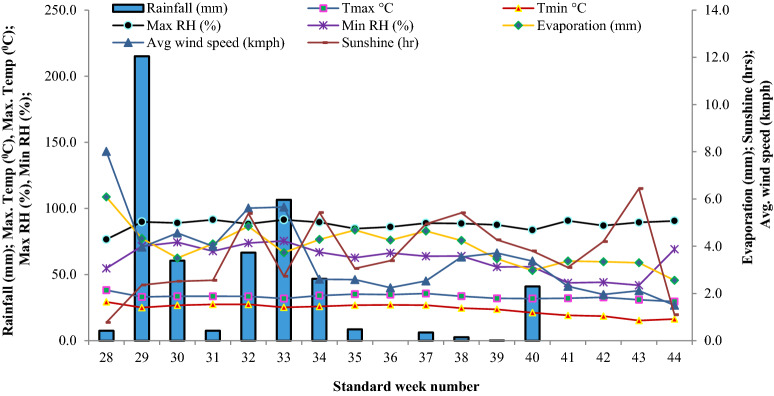
Weekly weather conditions during the cropping period of *Kharif* season maize in the Indo-Gangetic Plains, 2019.

A field experiment was carried-out in maize (*Zea mays* L.) for two years during *Kharif* 2018 and 2019 at Experimental Farm of ICAR-Indian Agricultural Research Institute, New Delhi, India [Latitude 28° 63′ N; Longitude 77° 15′ E; Altitude 228.6 m] under maize–wheat cropping system (MWCS). This experimental site is located in semi-arid sub-tropics having sandy-loam Alluvial soil belonging to Typic *Ustochrepts*. Climate is semi-arid with dry hot summers and cold winters with May and June as hottest months with mean daily maximum temperature varying from 40–46 °C (Figs. 10, 11).

Average annual rainfall is ~ 650 mm 80% of which is received through *'South-West Monsoons*' during July–September and the rest during *'Western Disturbances*' from December to February. Mean annual evaporation is ~ 850 mm. Physico-chemical analysis of composite soil samples (0–15 cm depth) was done at the start of the experiment using standard procedures (Table 4). Soil had pH 8.0, oxidizable soil organic–C 0.421%, alkaline KMnO_4_ oxidizable–N 137.9 kg ha^−1^, 0.5 M NaHCO_3_ extractable–P 12.9 kg ha^−1^ and 1 N NH4OAc extractable–K 302.8 kg ha^−1^.

**Table 4:** Initial fertility status of the experimental site.

S. No.	Particulars	Values	Method followed
1	**Mechanical analysis**		
	Sand (%)	64.9	Hydrometer method^122^
	Silt (%)	21.0	
	Clay (%)	14.1	
	Textural class	Sandy-loam	
2	**Physical properties**		
	Bulk density (Mg m^−3^)	1.57	Veihmeyer and Hendrickson^123^
3	**Chemical properties**		
	Organic carbon (%)	0.42	Walkley and Black method^124^
	Available N (kg ha^−1^)	137.9	Alkaline permanganate method^125^
	Available P (kg ha^−1^)	12.9	Olsen’s method^126^
	Available K (kg ha^−1^)	302.8	Flame photometer method^124^
	pH (1:2.5 soil: water ratio)	8.0	Beckman’s pH meter^127^
	EC (dSm^−1^) (1:2 soil: water ratio)	0.46	Richards^128^
4	**Biological properties**		
	Soil microbial biomass carbon (µg SMBC g soil^−1^)	183.4	Nunan et al.^129^
	Dehydrogenase activity (µg TPF g soil^−1^ day^−1^)	28.3	Casida et al.^130^
	Alkaline phosphatase activity (µg PNP g soil^−1^ h^−1^)	185.4	Tabatabai and Bremner^131^
	Acid phosphatase activity (µg PNP g soil^−1^ h^−1^)	29.2	Tabatabai and Bremner^131^

The experiment was laid-out in split-plot design with 3-replications and 20-treatment combinations comprised of 4 main-plot treatments i.e. crop establishment and tillage management (CETM) practices [M_1_: FBCT–FBCT (Flat bed–conventional tillage both in maize and wheat); M_2_: RBCT–RBZT (Raised bed–conventional tillage in maize and raised bed–zero tillage in wheat); M_3_: FBZT–FBZT (Flat bed–zero tillage both in maize and wheat); M_4_: PRBZT–PRBZT (Permanent raised bed–zero tillage both in maize and wheat)], and 5 P–fertilization practices in sub-plots [S_1_: P_100_ (100% P as basal); S_2_: P_50_ + 2FSP {50% P as basal (P_50_) + 2 foliar sprays of phosphorus (2FSP) as DAP (2%) at knee-high stage (KHS) and pre-tasseling stage (PTS) in maize and at tillering stage (TS) and pre-flowering stage (PFS) in wheat}; S_3_: P_50_ + PSB + AMF {P_50_ + PSB + AM-fungi (AMF)}; S_4_: P_50_ + PSB + AMF + 2 FSP (P_50_ + PSB + AMF + 2FSP at KHS and PTS in maize, and at TS and PFS in wheat); S_5_: P_0_ {100% N and K with no-P (P_0_) as control}]. Crop residues of preceding season wheat and maize crops were applied at 3 t ha^−1^ to all the ZT-plots except CT-plots after sowing of the succeeding crops of maize and wheat, respectively. In current study, ‘PMH-1’ high yielding single cross maize hybrid was used as the test cultivar being one of the most promising and popular cultivar of Indian IGPR. Hybrid ‘PMH-1’ was sown in gross plot size of 5.0 × 4.2 m at plant spacing of 60 × 25 cm using seed drill with seed at 20 kg ha^−1^ and fertilizer recommendation of N: P_2_O_5_: K_2_O at 150: 60: 40 kg ha^−1^ on 12th and 9th July and harvested on 29th and 24th October during *Kharif* 2018 and 2019, respectively. Whole K and whole treatment-wise fertilizer-P were applied as basal dose while N was applied in 3 equal splits (1/3rd as basal, 1/3rd top-dressed at KHS, 1/3rd top-dressed at PTS). Foliar P-fertilization was done at KHS and PTS using 2% DAP (Di-ammonium phosphate; 18% N and 46% P_2_O_5_) in 750 L water ha^−1^. Expect treatments, maize crop was grown using standard crop management practices^132^.

### Maize grain yield, protein content and protein yield

After harvesting, the maize crop from net-plots was sun-dried, threshed plot-wise, grains cleaned and sun-dried till 10% seed moisture was obtained. Grain yield (t ha^−1^) was estimated using standard procedures^132^. Nitrogen content (%) in maize grains was determined using standard procedure^132^. Protein content (%) in maize grains was calculated by multiplying grain-N content (%) by the factor 6.25 while protein yield (kg ha^−1^) in maize grains was calculated by using following formula:

$${\mathrm{Protein}}\,{\text{yield }}\,({\mathrm{kg}}/{\mathrm{ha}}) = \left(\frac{{\mathrm{Protein}}\,{\text{ content }}\,({{\%}})\times {\mathrm{ Grain}}\,{\text{yield} }\,({\mathrm{kg}}/{\mathrm{ha}})}{100}\right)$$

### Starch estimation

A grain sample of 0.4 g was homogenized in hot 80% ethanol to remove sugars. The residues retained after centrifugation were washed repeatedly with hot ethanol (80%) till the washing is colorless. The residues were dried and the extraction was done from the dried samples with the application of 5 mL water and 6.5 mL of percholoric acid (52%). The 0° C temperature was maintained for 20 min (min) and then samples were put under centrifugation at 10,000 rpm for 8 min. The supernatant was decanted and kept for starch estimation. The extraction was repeated 2–3 times for full and final extraction. With the addition of distilled water, final volume of the pooled-up supernatant was made to 100 mL. The 0.1 mL of supernatant was pipetted-out and the volume was made-up to 1.0 mL with distilled water. Similarly for reference, different aliquots of standard glucose solution were taken and volume was made-up to 1.0 mL using distilled water. The 4.0 mL of anthrone reagent was added to each tube and heated for 8 min in water bath. Intensity of color, green to dark green, was recorded at 630 nm^133^. The glucose concentration of the samples was determined using the calibration curve and the values obtained were multiplied by a factor 0.9 to quantify the starch content (%). Starch yield (kg ha^−1^) in maize grains was calculated by using following formula:

$${\mathrm{Starch}}\,{\text{ yield }}\,({\mathrm{kg}}/{\mathrm{ha}}) = \left(\frac{{\mathrm{Starch}}\,{\text{ content }}\,({\%})\times {\mathrm{ Grain}}\,{\text{ yield }}\,({\mathrm{kg}}/{\mathrm{ha}})}{100}\right)$$

### Amylose and amylopectin content

Maize grains from different plots were ground to make fine powder with particle size of 500 µ after milling. 100 mg of powdered samples was added with a mixture of ethanol and 1 M NaOH (1 mL + 10 mL) and was left as such overnight. Subsequently, distilled water was added to sample solution to make the final volume to 100 mL. An aliquot of 2.5 mL of extract was mixed with 20 mL distilled water and 3 drops of Phenolphthalein, where by the solution changes into pink-color. On addition of 0.1 M HCl drop by drop, the pink color disappears. To the treated sample, 1 mL of iodine reagent was added and volume was made-up to 50 mL by adding distilled water and then absorbance was recorded at 590 nm with reference to blank (1 mL iodine reagent diluted to 50 mL with distilled water). The amylose content in maize grains was determined using standard curve derived from potato amylose^134^. Standard amylose solution was prepared by dissolving 100 mg in 10 mL of 1 M NaOH and making up to 100 mL final volume. The amount of amylose in samples was determined by using standard curve prepared from amylase (0.2, 0.4, 0.6, 0.8 and 1.0 mL) against a blank for which dilute 1 mL of iodine reagent to 50 mL with water. The relevant calculations were done using following formula:

$${\mathrm{Amylose}}\,{\text{ content }}= \left({\mathrm{O.D.}} \times \frac{{\mathrm{Dilution}}\,{\text{ factor}}}{\mathrm{Slope}}\right)$$

Since, 2.5 mL of the test solution = x mg amylose; therefore, $100\,{\mathrm{ mL}}\,{\text{ contains }}= \left(\frac{{\mathrm{x}}}{2.5}\times 100\right)$.

The amylopectin content (%) in maize grains was determined by subtracting the amylose content from the total starch content^135^.

### Lysine and tryptophan estimation

The 5 mL papain solution was added to 100 g defatted maize grain sample and incubated at 65 °C overnight. It was cooled down to room temperature, centrifuged and decanted. Carbonate buffer (0.5 mL, pH 9.0) and copper phosphate suspension (0.5 mL) was added to 1 mL digest; after that the mixture was shaken for 5 min in a vortex mix and centrifuged. To 1 mL supernatant 0.1 mL of pyridine reagent was added, mixed well and shaken for 2 h. Then after adding 5 mL of 1.2 M HCl and mixing, extraction was done 3 times with 5 mL ethyl acetate, and ethyl acetate top layer was discarded. The absorbance of aqueous layer was read at 390 nm^136^. The standard lysine solution was prepared by dissolving 62.5 mg lysine mono hydrochloride in 50 mL carbonate buffer. For preparing a standard curve, 0.2, 0.4, 0.6, 0.8 and 1.0 mL of the standard lysine solution was pipetted out in different test tubes and final volume of 1 mL was made using carbonate buffer. Later, added 4 mL papain to each tube and mixed thoroughly. Now, 1 mL was pipetted out and 0.5 mL of amino acid mixture and 0.5 mL of copper phosphate suspension were added to it. Afterwards, 1 mL solution from each test tube was transferred to other test tubes and adding 0.5 mL amino acid mixture and 0.5 mL copper phosphate suspension to each one. The above steps were repeated as followed in case of samples and the absorbance of aqueous layer was read at 390 nm^136^. The lysine content in maize samples was determined from standard curve and results were expressed as g kg^−1^ dry matter.

For estimation of tryptophan, 15 mg defatted maize grain sample was taken in three different 50 mL conical flasks. In 2 flasks, 30 mg of *p*-dimethyl amino benzaldehyde was added. Third flask acted as the blank. To all the flasks, 9.5 M H_2_SO_4_ solution was added. The flasks were kept in dark for 20 h at 30 °C followed by addition of 0.1 mL of 0.045% NaNO_2_ solution to each flask. After mixing, the flasks were again kept for 30 min at room temperature. After centrifugation, the absorbance of blue color of the solution was measured at 660 nm^137^. A standard curve of tryptophan was prepared by taking various concentrations (10 to 60 µg mL) of standard tryptophan solution; the volume was made up to 0.6 mL by adding distilled water followed by addition of 9.4 mL of 9.5 M H_2_SO_4_ solution slowly and mixed gently. Same steps were followed for the standard solutions. Tryptophan content in the samples was determined from standard curve and expressed as µg g^−1^.

$${\mathrm{Tryptophan }}\,({\upmu {\text{g}}}/{\mathrm{g}})=\frac{{\upmu {\text{g}}\, {\text{tryptophan}}\, {\text{from}}\, {\text{standard}}\, {\text{curve}}}}{{\mathrm{Weight}}\, {\text{of}}\, {\text{grain}}\, {\text{sample }}\,({\mathrm{g}})}$$

### Oil content and oil yield

Oil content (%) in maize grains was determined by petroleum ether extraction in a Soxhlet apparatus for 16 h according to AOAC procedure 948.22^138^. Oil yield (kg ha^−1^) in maize grains was calculated by using following formula:

$${\mathrm{Oil}}\, {\text{yield }}\,({\mathrm{kg}}/{\mathrm{ha}}) = \left(\frac{{\mathrm{Oil}}\, {\text{content }}\,({\%})\times {\mathrm{ Grain}}\, {\text{yield }}\,({\mathrm{kg}}/{\mathrm{ha}})}{100}\right)$$

### Fatty acid analysis and fatty acid ratios

The 100 mg powdered maize grain samples were defatted with solvent mixture of Chloroform:Hexane:Methanol (8:5:2 v/v) for fatty acid analysis. The extracts were dried under a stream of nitrogen and fatty acids were converted into methyl-esters using 0.5 M KOH and 0.5 M HCl. Fatty acids were separated using Gas Chromatography-Mass Spectrometry (GC–MS) following the method as suggested by Kumar and Dhillon^139^. Separation of fatty acids viz. Palmitic acid, Stearic acid, Oleic acid and Linoleic acid was carried-out using HP Innowax capillary column (30 m × 0.32 m × 0.5 µm). The separated peaks were identified on the basis of retention time of standard fatty acid peaks and confirmed using GC–MS library. Besides fatty acid synthesis, different fatty acid ratios viz. ODR, MUFA: PUFA, SFA: UFA and PUFA: SFA were also worked-out using standard formulae^38^. These ratios were calculated excluding the linolenic acid because its contribution to total fatty acid composition was < 1% in maize grain oil.

$$\begin{aligned}{\mathrm{ODR}}&=\frac{{\%{\mathrm{C}}}_{18:2}}{\%{\mathrm{C}}_{18:1}+ \%{\mathrm{C}}_{18:2}}\\ {\mathrm{MUFA}}:{\mathrm{PUFA}}&=\frac{{\%\mathrm{C}}_{18:1}}{{\%\mathrm{C}}_{18:2}}\\ {\mathrm{SFA}}:{\mathrm{UFA}}&=\frac{{\%\mathrm{C}}_{16:0}+ {\%\mathrm{C}}_{18:0}}{{\%\mathrm{C}}_{18:1}+ {\%\mathrm{C}}_{18:2}}\end{aligned}$$

The P/S index is the ratio of polyunsaturated fatty acids (PUFA) and saturated fatty acids (SFA) and it was calculated by the following formula^140^:

$${\mathrm{P}}/{\mathrm{S}}\,{\text{index}}=\frac{{\mathrm{PUFA}}}{\mathrm{SFA}}$$

where ODR = Oleic desaturation ratio; MUFA = Monounsaturated fatty acid (Oleic acid); PUFA = Polyunsaturated fatty acid (Linoleic acid); SFA = Saturated fatty acid (Palmitic acid + Stearic acid); UFA = Unsaturated fatty acid; P/S index = PUFA/SFA ratio; C_18:1_ = Oleic acid; C_18:2_ = Linoleic acid; C_16:0_ = Palmitic acid; C_18:0_ = Stearic acid.

### Phosphorus content and its uptake in maize grains

Concentration of P in maize grains was determined by using the Vanadomolybdo-phosphoric acid yellow colour method at 420 nm wavelength on a UV–VIS spectrophotometer. From P content (%) in plants, P uptake (kg ha^−1^) was computed using the formula given below:

$${\text{P}}\;{\text{uptake}}\;{\text{in}}\;{\text{grains}}\;\left( {{\text{kg ha}}^{{ - {1}}} } \right) = \left[ {\% \;{\text{P}}\;{\text{in}}\;{\text{grains}} \times {\text{grain}}\;{\text{yield}}\;\left( {{\text{kg}}\;{\text{ha}}^{{ - {1}}} } \right)} \right]$$

### Statistical analysis

The data related to each parameter were analyzed as per the procedure of analysis of variance (ANOVA) to determine treatment effects through Tukey’s honestly significant difference test as a post hoc mean separation test (*p* < 0.05) by using SAS 9.1 software (SAS Institute, Cary, NC). Tukey’s procedure was used where ANOVA was found significant (Supplementary Tables S1 and S2). A two-dimensional heatmap with hierarchical clustering of treatment-by-traits was drawn using R-software package ‘gplots’ developed by Warnes et al.^141^. To reduce the complexity of relationship, a data reduction technique was performed using principal component analysis (PCA) implemented in the R package ‘Factoextra’ and ‘FactoMineR’, and thereby resulting PC scores were plotted^142,143^.

### Research involving plants

It is stated that the current experimental research on the plants comply with the relevant institutional, national, and international guidelines and legislation. It is also stated that the appropriate permissions has been taken wherever necessary, for collection of plant or seed specimens. It is also stated that the authors comply with the ‘IUCN Policy Statement on Research Involving Species at Risk of Extinction’ and the ‘Convention on the Trade in Endangered Species of Wild Fauna and Flora’.

## Supplementary Information

Supplementary Information.

## Abbreviations

AMF: Arbuscular mycorrhizal fungi
CA: Conservation agriculture
CETM: Crop establishment and tillage management
CPTs: Clean production technologies
CRR: Crop residue retention
C_18:1_: Oleic acid
C_18:2_: Linoleic acid
C_16:0_: Palmitic acid
C_18:0_: Stearic acid
CT: Conventional tillage
DAP: Di-ammonium phosphate
EC: Electrical conductivity
FBCT: Flat bed–conventional tillage
FBZT: Flat bed–zero tillage
2FSP: Two foliar sprays of phosphorus
ha: Hectare
IGPR: Indo-Gangetic Plains Region
K: Potassium
KHS: Knee-high stage in maize
m: Meter
m ha: Million hectares
Mt: Million tonnes
MWCS: Maize–wheat cropping system
MUFA: Monounsaturated fatty acid
N: Nitrogen
ODR: Oleic desaturation ratio
P: Phosphorus
P_0_: No-phosphorus
P_50_: 50% recommended dose of P (as basal)
P_100_: 100% recommended dose of P (as basal)
PUE: Phosphorus-use efficiency
PRBZT: Permanent raised bed–zero tillage
PSB: Phosphorus solubilizing bacteria
PUFA: Polyunsaturated fatty acid
PUE: Phosphorus-use efficiency
P/S index: PUFA/SFA ratio
RBCT: Raised bed–conventional tillage
RWCS: Rice–wheat cropping system
SFA: Saturated fatty acid
SOC: Soil organic carbon
t: Tonnes
TS: Tillering stage in wheat
UFA: Unsaturated fatty acid
ZT: Zero tillage
