# Supplementary material for: Double zero tillage and foliar phosphorus fertilization coupled with microbial inoculants enhance maize productivity and quality in a maize–wheat rotation
Source: Sci Rep. 2022 Feb 24;12:3161. doi: 10.1038/s41598-022-07148-w (PMC8873388; doi:10.1038/s41598-022-07148-w)
Supplement: Supplementary file 1 — Supplementary Information. [file 41598_2022_7148_MOESM1_ESM.docx]

**Double zero tillage and foliar phosphorus fertilization coupled with microbial inoculants enhance maize productivity and quality in a maize-wheat rotation**

**Harish M. N.^1,†^, Anil K. Choudhary^1,2,†,*^, Sandeep Kumar^3^, Anchal Dass^1^, V. K. Singh^1,4^, V. K. Sharma^1^, Varatharajan T.^1^, M. K. Dhillon^1^, Seema Sangwan^5^, V. K. Dua^2^, Nitesh S. D.^6^, Bhavya M.^7^, S. Sangwan^1^, Shiv K. Prasad^1^,** **Adarsh Kumar^1,8^, S.K. Rajpoot^1,9^, Gaurendra Gupta^1,10^, Prakash Verma^1,11^, Anil Kumar^12^ & S. George^13^**

^1^ICAR–Indian Agricultural Research Institute, New Delhi 110 012, India. ^2^ICAR–Central Potato Research Institute, Shimla, Himachal Pradesh 171 001, India. ^3^ICAR–National Bureau of Plant Genetic Resources, New Delhi 110012, India. ^4^ICAR–Central Research Institute for Dryland Agriculture, Hyderabad 500 059, India. ^5^CCS Haryana Agricultural University, Hisar, Haryana 125 004, India. ^6^CSA University of Agriculture & Technology, Kanpur, Uttar Pradesh 208 002, India. ^7^University of Agricultural & Horticultural Sciences, Shivamogga, Karnataka 577 204, India. ^8^ICAR-**Indian Institute of Seed Sciences,** Kushmaur, Mau, Uttar Pradesh 275 103, India. ^9^Institute of Agricultural Sciences, Banaras Hindu University, Varanasi, Uttar Pradesh 221 005, India. ^10^ICAR–Indian Grassland and Fodder Research Institute, Jhansi, Uttar Pradesh 284 003, India. ^11^ICAR-National Dairy Research Institute, Karnal, Haryana 132 001, India. ^12^GAD Veterinary and Animal Sciences University, Farm Science Centre, Tarn Taran, Punjab 143 412, India. ^13^ICAR-Indian Institute of Horticultural Research, Farm Science Centre, Gonikoppal, Karnataka 571213, India.

*Corresponding author E-mail: anilhpau2010@gmail.com.

**†Joint first Authors.**

**Supplementary Table S1**: Analysis of variance of various parameters that were measured in the maize.

| **Parameters** | **df** | **Grain yield** | | | **Starch** | | | **Starch yield** | | | **Amylose** | | | **Amylopectin** | | | **Grain P uptake** | | |
| --- | --- | --- | --- | --- | --- | --- | --- | --- | --- | --- | --- | --- | --- | --- | --- | --- | --- | --- | --- |
|  |  | **2018** | **2019** | **Mean** | **2018** | **2019** | **Mean** | **2018** | **2019** | **Mean** | **2018** | **2019** | **Mean** | **2018** | **2019** | **Mean** | **2018** | **2019** | **Mean** |
| Replications | 2 | **-** | **-** | **-** | **-** | **-** | **-** | **-** | **-** | **-** | **-** | **-** | **-** | **-** | **-** | **-** | **-** | **-** | **-** |
| CETM practices | 3 | * | * | * | ns | ns | ns | * | * | * | ns | ns | ns | ns | ns | ns | * | * | * |
| P–fertilization practices | 4 | * | * | * | * | * | * | * | * | * | * | * | * | * | * | * | * | * | * |
| CETM × P–fertilization | 12 | * | * | * | ns | ns | ns | ns | ns | ns | ns | ns | ns | ns | ns | ns | * | * | * |

**Note:** ns: non-significant; *: Significant.

**Supplementary Table S2**: Analysis of variance of various parameters that were measured in the maize.

| **Parameters** | **df** | **Protein content** | | | **Protein yield** | | | **Lysine content** | | | **Tryptophan** | | | **Oil content** | | | **Oil yield** | | |
| --- | --- | --- | --- | --- | --- | --- | --- | --- | --- | --- | --- | --- | --- | --- | --- | --- | --- | --- | --- |
|  |  | **2018** | **2019** | **Mean** | **2018** | **2019** | **Mean** | **2018** | **2019** | **Mean** | **2018** | **2019** | **Mean** | **2018** | **2019** | **Mean** | **2018** | **2019** | **Mean** |
| Replications | 2 | **-** | **-** | **-** | **-** | **-** | **-** | **-** | **-** | **-** | **-** | **-** | **-** | **-** | **-** | **-** | **-** | **-** | **-** |
| CETM practices | 3 | ns | * | * | * | * | * | ns | ns | ns | ns | ns | ns | ns | ns | ns | * | * | * |
| P–fertilization practices | 4 | * | * | * | * | * | * | * | * | * | * | * | * | * | * | * | * | * | * |
| CETM × P–fertilization | 12 | ns | ns | ns | ns | * | ns | ns | ns | ns | ns | ns | ns | ns | ns | ns | ns | ns | ns |

**Note:** ns: non-significant; *: Significant.
